# Association of Variants in *IL-1RN* (rs2234663) and *IL-1β* (rs1143627, rs16944) and Interleukin-1β Levels with Colorectal Cancer: Experimental Study and In Silico Analysis

**DOI:** 10.3390/genes15121528

**Published:** 2024-11-27

**Authors:** Martha Patricia Gallegos-Arreola, Asbiel Felipe Garibaldi-Ríos, Itzae Adonaí Gutiérrez-Hurtado, Guillermo Moisés Zúñiga-González, Luis E. Figuera, Belinda Claudia Gómez-Meda, Ana María Puebla-Pérez, José Elías García-Ortiz, Jorge I. Delgado-Saucedo, Paola Beatriz Castro-García, María de Jesús Rentería-Ramírez, Blanca Miriam Torres-Mendoza

**Affiliations:** 1 División de Genética, Centro de Investigación Biomédica de Occidente, Centro Médico Nacional de Occidente, Instituto Mexicano del Seguro Social, Guadalajara 44340, Jalisco, Mexico; asbiel.garibaldi4757@alumnos.udg.mx (A.F.G.-R.); luisfiguera@yahoo.com (L.E.F.); jose.garciaor@imss.gob.mx (J.E.G.-O.); gato6613@gmail.com (M.d.J.R.-R.); 2 Doctorado en Genética Humana, Centro Universitario de Ciencias de la Salud, Universidad de Guadalajara (UdeG), Guadalajara 44340, Jalisco, Mexico; 3 Departamento de Biología Molecular y Genómica, Instituto de Genética Humana “Dr. Enrique Corona Rivera”, Centro Universitario de Ciencias de la Salud, Universidad de Guadalajara, Guadalajara 44340, Jalisco, Mexico; itzae.gutierrez@academicos.udg.mx (I.A.G.-H.); belinda.gomez@academicos.udg.mx (B.C.G.-M.); 4 División de Medicina Molecular, Centro de Investigación Biomédica de Occidente, Centro Médico Nacional de Occidente, Instituto Mexicano del Seguro Social, Sierra Mojada 800, Col. Independencia, Guadalajara 44340, Jalisco, Mexico; mutagenesis95@hotmail.com; 5 Laboratorio de Inmunofarmacología, Centro Universitario de Ciencias Exactas e Ingenierías, Universidad de Guadalajara (UdeG), Guadalajara 44430, Jalisco, Mexico; ana.puebla@academicos.udg.mx (A.M.P.-P.); jorge.delgado@academicos.udg.mx (J.I.D.-S.); paola.castro@academico.udg.mx (P.B.C.-G.); 6 Laboratorio de Inmunodeficiencias y Retrovirus Humanos, División de Neurociencias, Centro de Investigación Biomédica de Occidente, Centro Médico Nacional de Occidente, Guadalajara 44340, Jalisco, Mexico; blanca.torresm@imss.gob.mx; 7 Departamento de Clínicas Médicas, Centro Universitario de Ciencias de la Salud, Universidad de Guadalajara, Guadalajara 44340, Jalisco, Mexico

**Keywords:** colorectal neoplasms, interleukin-1, polymorphism, genetic, cytokines

## Abstract

Background/Objectives. Colorectal cancer (CRC) is a multifactorial disease where the inflammatory state is crucial. This study analyzes the association of the IL-1RN (rs2234663) and IL-1β (rs1143627, rs16944) variants and IL-1β levels with CRC. Methods. This study included 230 CRC patients and 256 controls. Genotypes were determined by PCR and plasma IL-1β levels by ELISA. RegulomeDB analyzed the variants’ functional impacts, while OncoDB assessed *IL-1β* and *IL-1RN* expression’s influence on CRC. Results. The A1A1 genotype and dominant pattern of the rs2234663 variant were risk factors for CRC, whereas the A1A2 genotype showed a protective effect. The TC genotype of the rs1143627 variant and the T allele of rs16944 were associated with increased risk, whereas the C allele had a protective effect. The A1A1 genotype was associated with stage I–II CRC diagnosis, while the A2A2 genotype was associated with stage III–IV and ethanol consumption. The CC genotype of rs1143627 was associated with people younger than 50 years and tobacco use, and the TCCC genotype was related to stage III–IV stages and metastasis and hemorrhoids (*p* < 0.05). IL-1β levels were not associated with CRC. In silico analysis revealed that the variants are in located in important regions regulatory of genes. Elevated *IL-1B* and *IL-1RN* mRNA levels were found in CRC, linked to clinicopathological features of the disease. Conclusions. The analyzed variants are associated with CRC and may influence gene regulation by being located at critical sites of key genetic regulators.

## 1. Introduction

Colorectal cancer (CRC) is characterized by the uncontrolled growth of cancer cells in the colon and rectum [1]. Recent data from GLOBOCAN indicate that it is the third most commonly diagnosed neoplasm worldwide and the second leading cause of cancer mortality [2]. Although CRC is a multifactorial disease, genetic aspects contributing to chronic inflammation, a key factor in its etiology, are of great relevance due to their role in carcinogenesis [1,3,4,5]. In this context, the *IL-1RN* and *IL-1β* genes play critical roles in the regulation of the inflammatory response, with genetic variants in these genes potentially influencing the levels of this cytokine and increasing the risk of CRC.

The *IL-1RN* and *IL-1β* genes play critical roles in the regulation of the inflammatory response. *IL-1RN* encodes the interleukin-1 antagonist receptor, a molecule that inhibits the activity of the proinflammatory cytokines IL-1α and IL-1β, thus modulating inflammation [6]. Variants in *IL-1RN*, such as rs2234663, may alter its function and influence susceptibility to inflammatory diseases and CRC [7,8,9]. On the other hand, *IL-1β* encodes interleukin-1β, a potent proinflammatory cytokine involved in the promotion of cell proliferation, angiogenesis, and the inhibition of apoptosis [10], all processes that facilitate cancer development and progression. Genetic variants in *IL-1β*, such as rs1143627 and rs16944, have been associated with various inflammatory diseases [11] and breast [12] and cervical [13] cancers.

According to the NCBI SNP database, the variants analyzed in this study are located in the regulatory regions of the genes; the rs2234663 variant is a multiallelic insertion-deletion (INDEL) of VNTR (variable number tandem repeat) type, located in intron 2 of the *IL-1RN* gene [14]. On the other hand, the rs1143627 variant is located in the 5′ region of the *IL-1β* gene and corresponds to a single nucleotide variant (SNV) of transition type (G > A), where the G allele presents a global frequency of 0.528 and the A allele a frequency of 0.472 [15]. In addition, the variant rs16944, also located in the 5′ UTR region of the *IL-1β* gene, is another transition-type SNV (A > G), with a worldwide frequency of the A allele of 0.509 and of the G allele of 0.491 [16].

Thus, understanding how these genetic variants influence inflammation and CRC pathogenesis is critical for the development of preventive and therapeutic strategies.

## 2. Materials and Methods

### 2.1. Study Population

Genomic DNA was isolated from peripheral blood samples collected from 230 individuals diagnosed with CRC and 256 healthy subjects who served as the reference group. Participants self-reported Mexican ancestry for at least three generations to ensure population homogeneity. The procedure involved lysing leukocytes using a lysis buffer, followed by protein digestion with Proteinase K and DNA precipitation through a salting-out method, ensuring high-quality DNA for downstream analyses. The CRC study group included men and women from the Mexican population, over 18 years of age, with a clinical and pathological diagnosis of CRC. The reference group was composed of men and women over 18 years of age, healthy donors, and volunteers from the Mexican population.

This study was conducted following the Declaration of Helsinki, which establishes ethical principles for medical research involving human subjects, including informed consent, protection of privacy, and well-being of the participants. All subjects signed an informed consent form. The protocol was approved by the local ethics committee (registration 2015-1305-3, 2024-1305-039) of the Centro de Investigación Biomédica de Occidente, Instituto Mexicano del Seguro Social (CLIES #1305), complying with all applicable ethical and legal regulations.

### 2.2. Genotyping of Variants

Genotyping of the variants was performed by polymerase chain reaction (PCR) followed by enzymatic digestion. The PCR mixture had a total volume of 15 μL and included 0.25 mM dNTPs (Invitrogen, Carlsbad, CA, USA), 5 pmol of each primer, 3.0 mM MgCl_2_, 1 μL of DMSO, 2.5 U of Taq polymerase (Invitrogen, Carlsbad, CA, USA) and 50 ng of genomic DNA. Amplification was performed at an alignment temperature of 56 °C for the IL-1RN variant (rs2234663), 58 °C for the rs1143627 variant, and 55 °C for the rs16944 variant.

Primers used for each variant were as follows: for rs2234663, 5′-CTC AGC AAC ACT CCT AT-3′ (sense) and 5′ TCC TGG TCT GCA GGT AA 3′ (antisense). The rs1143267 variant used the primers 5′-AGGCAATAGGTTTTTTGAGGGCCAT-3′ (sense) and 5′-TCCTCCCTGCTCCCGGATTTCCG-3′ (antisense), while the rs16944 variant amplified the primers 5′-AGAAGACCCCCCCCTCGGAACC-3′ (sense) and 5′ TTGGGGGGACACACAAGCATCAAGG-3′ (antisense).

**Figure 1:**
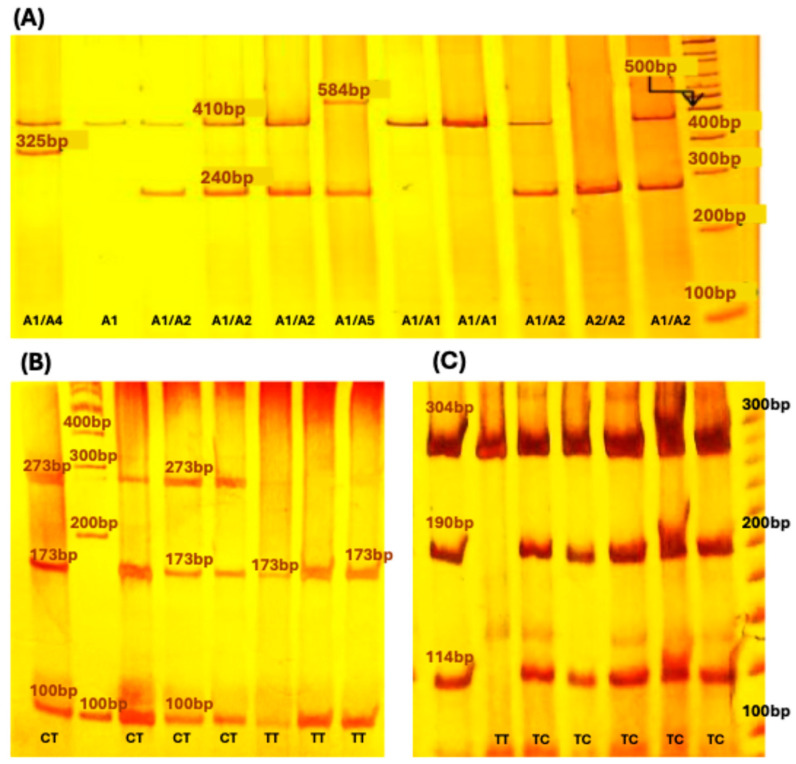
(**A**) Electrophoretic bands corresponding to the alleles of the rs2234663 variant identified on 6% (29:1) polyacrylamide gels stained with silver nitrate: allele 1 (410 bp, 4 repeats), allele 2 (240 bp, 2 repeats), allele 3 (500 bp, 5 repeats), allele 4 (325 bp, 3 repeats), and allele 5 (584 bp, 6 repeats). (**B**) Genotypes of rs1143627 digested with *AluI*: TT (173 and 100 bp) and heterozygous CT (100, 173, and 273 bp). (**C**) Genotypes of rs16944 digested with *AvaI*: TT (304 bp) and heterozygous TC (114, 190, and 304 bp).

Genotypes of the rs2234663 variant were identified on 6% (29:1) polyacrylamide gels stained with silver nitrate. The alleles were as follows: allele 1 of 410 bp (A1 = 4 repeats of 86 bp), allele 2 of 240 bp (A2 = 2 repeats), allele 3 of 500 bp (A3 = 5 repeats), allele 4 of 325 bp (A4 = 3 repeats), and allele 5 of 584 bp (A5 = 6 repeats) (Figure 1A). PCR products for IL-1β variants were digested with variant-specific restriction enzymes: AluI for rs1143627, where the TT genotype (polymorphic) was identified by 2 bands of 173 and 100 bp, the wild-type CC by a single band of 273 bp, and the heterozygote by 3 bands of 100, 173, and 273 bp (Figure 1B). The AvaI restriction enzyme was used for rs16944, with the TT genotype showing a single band of 304 bp, the CC genotype 2 bands of 114 and 190 bp, and the heterozygote 3 bands of 114, 190, and 304 bp (Figure 1C). The resulting fragments were separated on 6% polyacrylamide gels (29:1) and visualized by silver staining. To ensure accuracy, 10% of the reactions were repeated.

For the rs2234663 variant, no restriction enzymes were required, since the alleles were identified based on their distinct molecular weights. This allowed for direct visualization of the different alleles by comparing the size of the PCR products, where each allele exhibited a unique banding pattern corresponding to the number of repeats in the fragment. In contrast, the restriction enzymes, AluI and AvaI, were necessary for rs1143627 and rs16944 because they recognize specific recognition sites in the amplified fragments. The use of these restriction enzymes ensured precise genotyping by cleaving the amplified products only at the target sequences, which helped to accurately identify the genetic variants of *IL-1β*.

### 2.3. Quantification of Plasma IL-1β Levels

Plasma levels of IL-1β were measured using an ELISA technique following the manufacturer’s instructions (R&D Systems Inc., Minneapolis, MN, USA), with the ELISA kit catalog number QLB00B. Absorbance was read at 450 nm using a microplate reader (iMark™ Microplate Absorbance Reader, BIORAD, Hercules, CA, USA).

### 2.4. In Silico Analysis

To assess the biological and molecular impact of the variants studied, the RegulomeDB platform [17] (http://regulomedb.org, accessed on 15 July 2024) was used to investigate how these variants might influence gene regulation. In addition, through the OncoDB platform [18] (http://oncodb.org, accessed on 17 July 2024), we analyzed *IL-1RN* and *IL-1β* mRNA expression in colon cancer (COAD) and rectal cancer (READ) samples.

### 2.5. Statistical Analysis

The allelic and genotypic frequency of the variants was determined by direct counting, which allowed us to obtain an accurate estimate of the distribution of alleles in the population studied. To assess the Hardy–Weinberg equilibrium, the chi-square test was used to compare the expected genotypes with the observed ones.

SPSS 24 software (IBM Corp., Armonk, NY, USA) was used to perform comparisons of genotypic frequencies between the CRC group and the reference group. In addition, odds ratios were calculated to examine the relationship between genotypic frequencies and clinicopathologic characteristics of the CRC group, providing valuable information on the association between genetic variants and disease progression. A value of *p* < 0.05 was considered significant for all statistical tests performed. For in silico analyses, *p*-values < 0.05 were considered to compare expression patterns between study groups.

## 3. Results

### 3.1. Sociodemographic and Clinicopathological Characteristics of the Study Groups

When analyzing the sociodemographic characteristics of the study groups, it was observed that the average age at diagnosis of colorectal cancer (CRC) was 59.4 ± 12.1 years in the study population, compared to 59.0 ± 11.9 years in the reference group, though no significant differences were observed (*p* > 0.05) (Table 1). The gender distribution was similar, with 49% of CRC patients being female and 51% male. Tobacco and alcohol consumption were significantly associated with CRC, with odds ratios of 3.3 (*p* < 0.001) and 4.4 (*p* < 0.0001), respectively (Table 1).

When the clinicopathological characteristics were analyzed in the CRC group, we observed that 35% had colon cancer and 65% had rectal cancer. Most were diagnosed at advanced stages (III–IV) (84%). Regarding the response to chemotherapy, 36% of patients responded positively, while 64% did not. In laboratory tests, 67% of patients had elevated carcinoembryonic antigen (CEA) levels (Table 2).

### 3.2. Allelic and Genotypic Frequencies of the Variants

When analyzing the allelic and genotypic frequencies of the variants among the study groups, we observed that the A1A1 genotype (OR 1.5, 95% CI 1.50–1.04, *p* = 0. 027) and the dominant pattern (OR 1.95, 95% CI 1.14–3.32, *p* = 0.013) of the rs2234663 variant were associated as risk factors for CRC. On the other hand, the A1A2 genotype was identified as a protective factor (OR 0.6, 95% CI 0.41–0.88, *p* = 0.008). In addition, the TC genotype of the rs1143627 variant was observed as a risk factor for CRC (OR 1.5, 95% CI 1.1–2.2, *p* = 0.02). As for the rs16944 variant, the T allele was associated as a risk factor for CRC (OR 1.6, 95% CI 1.2–2.0, *p* = 0.003), whereas the C allele was identified as a protective factor (OR 0.6, 95% CI 0.5–0.8, *p* = 0.003) (Table 3).

When we compared genotypes with the clinicopathologic characteristics of CRC patients, we observed that the A1A1 genotype of the rs2234663 variant was associated with the early stages (I–II) in those CRC patients diagnosed at less than 5 years (OR 2.6, 95% CI 1.1–5.8, *p* = 0.016), as well as with the A2A2 genotype in advanced stages (III–IV), and with alcohol-consuming patients (OR 10, 95% CI 1.3–38.1, *p* = 0.007); however, the confidence intervals were wide. The CC genotype of the rs1143627 variant was associated with ages younger than 50 years (OR 2.3, 95% CI 1.1–5.6, *p* = 0.046). Furthermore, the CT genotype of the rs16944 variant was associated with hemorrhoids/diverticula (OR 2.7, 95% CI 1.1–7.1, *p* = 0.041), while the TT genotype was associated with gastric toxicity (OR 1.8, 95% CI 1.1–3.3, *p* = 0.040). Finally, the TCCC genotype correlated with multiple factors in stage III–IV, including lymph-node metastases (OR 3.1, 95% CI 1.3–7.9, *p* = 0.01) (Table 4).

When analyzing haplotypic frequencies, we observed that haplotype A1TC presented the highest frequency, with 0.21 in both the CRC group and the reference group. Haplotype A1CT followed this in the CRC group (0.15), and haplotype A2TC presented the same way in the reference group (0.22). No statistically significant differences were found between the study groups. The linkage disequilibrium analysis obtained a value of D’ = 0.5 (Table 5).

**Figure 2:**
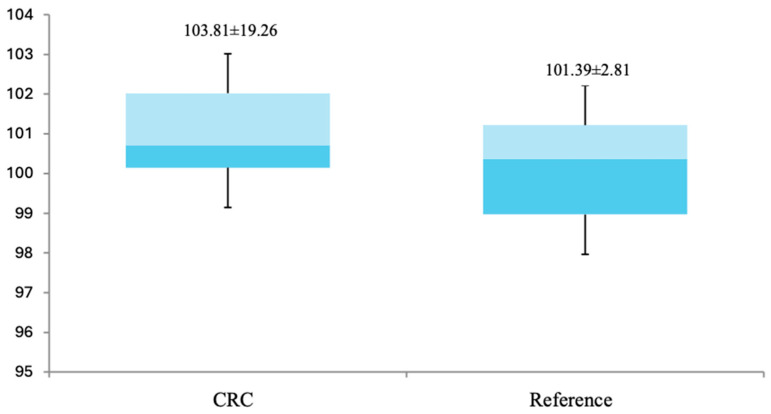
Plasma levels of IL-1β. No significant differences in mean IL-1β levels were observed between the CRC group and the reference group (*p* > 0.05).

Comparative analysis of plasma IL-1β levels between CRC patients and the reference group revealed no significant differences. Likewise, the comparison of plasma values between stage I–II and III–IV patients also showed no significant differences (Figure 2 and Table 6).

### 3.3. In Silico Analysis

#### 3.3.1. Gene Regulation Mediated by the Studied Variants

**Figure 3:**
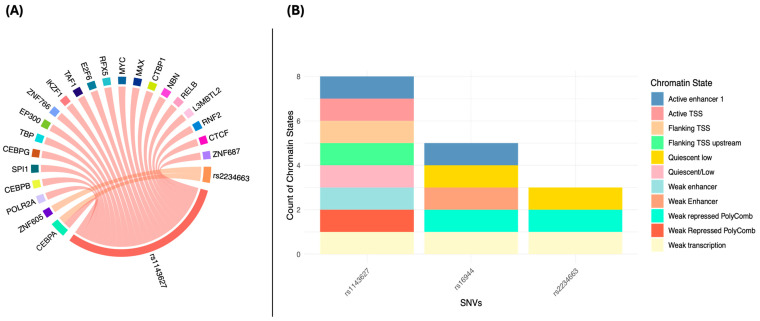
Regulatory elements (**A**) targeting the genomic region of the variants. The rs1143627 variant is located in a region harboring numerous *IL-1β* regulatory elements, including transcription factors. Similarly, rs2234663 is located in regions where CEBPA and ZNF605 play regulatory roles on *IL-1RN.* (**B**) The chromatin state in the genomic region of the variants. It is observed that the variants are located in regions with a chromatin state associated with regulatory functions, indicating that they could influence the regulation of gene expression.

Results obtained from the RegulomeDB database for the rs1143627, rs16944, and rs2234663 variants show different rankings of functional evidence. The rs1143627 variant has a score of 0.76 and a range of 1b, corresponding to evidence for quantitative trait expression (eQTL/caQTL), transcription factor (TF) binding with any motif, a footprint, and a chromatin accessibility peak. The rs16944 variant has a range of 1f and a score of 0.19549, indicating that it has evidence of eQTL/caQTL, TF binding, and a chromatin accessibility peak, but no specific motif. Finally, the rs2234663 variant is classified with a score of 4 and a score of 0.60906, indicating that there is evidence of TF binding and a chromatin accessibility peak (Figure 3).

#### 3.3.2. mRNA Analysis

**Figure 4:**
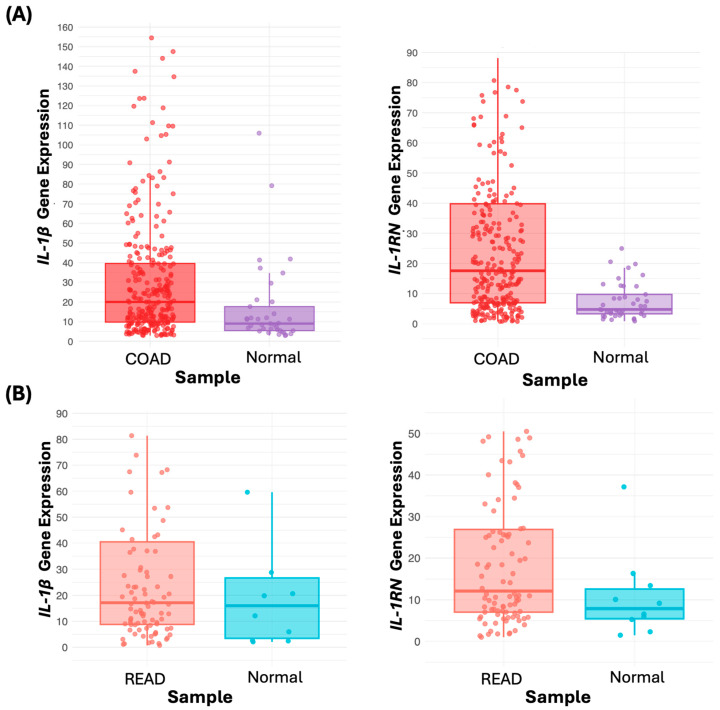
Expression of *IL-1β*, and *IL-1RN* in COAD (**A**). Statistically significant differences (*p* < 0.05) were identified in *IL-1β* expression in COAD (median = 20.5) versus normal tissue (median = 8.4), as well as in *IL-1RN* in COAD (median = 17.6) compared to normal tissue (median = 4.7). In READ (**B**), significant differences were observed in *IL-1RN* expression (median = 12.1) versus normal tissue (median = 7.9), while no differences were found in *IL-1β* expression between READ tissues and normal tissue (*p* > 0.05). (Outliers were removed from the plot to improve the Y-axis visualization, but the median values were calculated considering all data, including outliers).

When analyzing the mRNA expression of *IL-1RN* and *IL-1β*, we observed that both genes are overexpressed in CRC. IL-1β shows a median expression of 20.5 in colon cancer (COAD) versus 8.4 in the normal colon (log FC 1.29, *p* = 6.8 × 10^−4^). On the other hand, *IL-1RN* shows a median of 17.6 in COAD compared to 4.7 in the unaltered colon (log FC 1.90, *p* = 1 × 10^−12^) (Table 7, Figure 4A). In the case of rectal cancer (READ), significantly higher *IL-1RN* gene expression was observed, with a median of 12.1 in READ versus 7.9 in the normal rectum (log FC 0.62, *p* = 1.5 × 10^−2^) (Table 7, Figure 4B).

**Figure 5:**
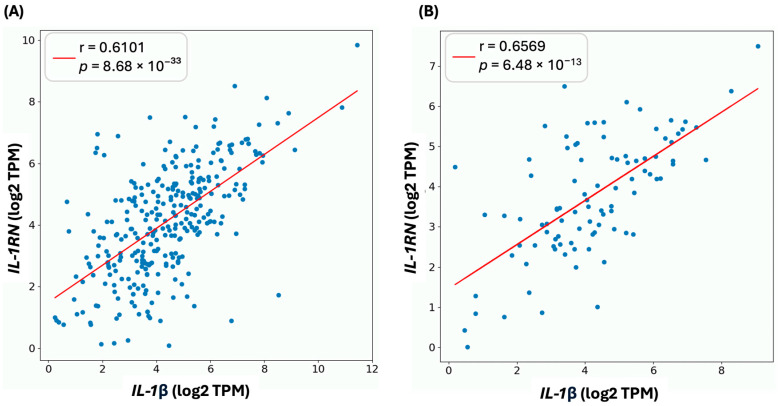
Correlation of expression between *IL-1β* and *IL-1RN* in COAD (**A**) and READ (**B**). In COAD and READ, a mutual correlation is observed between the expression of *IL-1β* and *IL-1RN*.

In addition, we observed a positive correlation in the co-expression of IL-1β, and IL-1RN in COAD (r = 0.6101, *p* = 8.68 × 10^−33^) (Figure 5A) and READ (r = 0.6569, *p* = 6.48 × 10^−13^) (Figure 5B).

**Figure 6:**
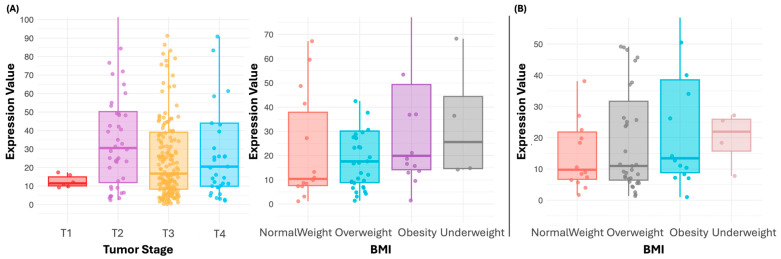
Expression of *IL-1β* in the pathological T stage of COAD and by BMI in READ (**A**), and *IL-1RN* by BMI in READ (**B**). Differences in *IL-1β* expression were observed when comparing T status in COAD, with higher expression in T2 and lower in T1 (*p* < 0.05). In READ, *IL-1β* showed higher expression in underweight, overweight, and obese individuals, compared to those with normal weights (*p* < 0.05). *IL-1RN* showed significantly higher expression in underweight compared to normal-weight individuals in READ (*p* < 0.05). Outliers were removed from the plot to improve the Y-axis visualization, but the median values were calculated considering all data, including outliers.

Comparing clinicopathological characteristics with IL-1β mRNA expression, it was observed that the expression of this gene is associated with the pathological T stage in COAD. Higher expression was found in the T2 stage (median = 30.6), while lower expression was observed in the T1 stage (median = 11.5) (*p* = 1.7 × 10^−3^) (Figure 5A). The median expression for T3 was 16.7, and for T4, it was 20.5. On the other hand, in READ, higher expression was also associated with being underweight, obesity, and being overweight (median = 25.6, 19.9 and 17.6, respectively, with lower expression observed in normal weight (median = 10.4) (Figure 6A, Table 8) (*p* = 2 × 10^−2^).

In addition, *IL-1RN* was also associated with body mass index (BMI) in READ, with higher expression in individuals who were underweight, obese, or overweight (median = 21.9, 13.4, and 11, respectively) and lower expression in individuals with normal weights (median = 9.8) (*p* = 3.4 × 10^−2^) (Figure 6B, Table 8).

## 4. Discussion

### 4.1. General Characteristics of the Study Groups

In our analysis regarding the clinical characteristics of the patients, we observed that the age at diagnosis of colorectal cancer (CRC) was usually older than 50 years, which has been described in other populations [19]. The frequency of CRC occurrence in men and women showed a slight increase in men (51%) compared to women. In this regard, the most recent reports provided by GLOBOCAN [2] indicate that the frequency of CRC in five continents is similar in both genders, although slightly higher in men than in women [20]. In addition, tobacco and alcohol consumption were found to be higher in the patient group; in this context, it has been established that alcohol and tobacco consumption are risk factors for the development of various pathologies, including CRC [21].

### 4.2. Clinical Characteristics of the Group of Patients

When we compared the clinicopathological characteristics of the study patients, we observed that the majority of the tumors were located on the left side, mainly affecting the rectum. This was similar to the characteristics described in the literature, where it was estimated that 60% of tumors were located on the left side of the rectum [19]. Many CRC patients were diagnosed at advanced stages (III, and IV); in this sense, it has been described that the diagnosis of patients with CRC in advanced stages is due to the lack of programs for early detection of CRC, as well as the absence of specific symptoms of the disease, which favors diagnosis in advanced stages [20,21]. In the present study, the presence of metastases, elevated levels of embryonic carcinoma (CEA), and non-response to chemotherapy were also observed in high frequency. Studies in the literature have shown that the presence of metastases and high levels of CEA are considered proportional to tumor development [22,23].

### 4.3. Variant in the IL-1RN Gene

The *IL-1RN* antagonist is known to function as a cytosine by competitively inhibiting the binding of IL1-β to its receptor, preventing the activation of the signaling cascade and the inflammatory process [24]. IL-1RN levels increase during the inflammatory process to terminate the acute phase of the inflammatory process [25]. The rs2234663 variant studied in this gene is known as 86bp VNTR, located in intron 2 of the gene and actively functioning in its regulation. In this study, the A1 allele was the most frequent and the A2 allele the least frequent; these results were congruent with those described in the literature [26,27]. Regarding the A3, A4, and A5 alleles, the frequency observed in both study groups was less than 1%, which is consistent with that described in the literature [28]. In the association of the polymorphism in intron 2 of the *IL-1RN* gene with CRC, we observed that both the A1 allele and the A1A1 genotype were associated with CRC. It has been observed that carriers of long alleles such as A1, A3, A4, and A5 have lower levels of IL-1RN compared with carriers of the A2 allele, so it is thought that reduced levels of IL-1RN favor a more severe inflammatory reaction, which could influence the development of cancer [29,30]

It has been observed that 89% of patients with stage II CRC were homozygous for the A1 allele [30]. Additionally, it was observed that a higher frequency of carriers of the A1 allele were in the early stages of CRC [30,31]. On the other hand, one study has associated A2 with increased IL-1RN production [32], so it could be expected that the A2A2 genotype would be associated with decreased risk of CRC development; however, this association was not demonstrated in the present study. Additionally, when comparing the genotypes only in the group of patients according to early (I and II) and advanced (III and IV) stages, it was observed that the A2A2 genotype was associated with the advanced stages of CRC. A possible explanation for this could be that individuals homozygous for the A2 allele experience a shorter CRC recurrence period (5.7 years) compared to homozygotes for the A1 allele (10.7 years) [30]. This shorter recurrence period suggests a faster progression of cancer, leading to diagnosis at more advanced stages. One plausible explanation for why the A2A2 genotype might be linked to advanced CRC is that the A2 allele may interact with other genetic polymorphisms that increase IL-1β production, which plays a significant role in the inflammatory pathways involved in cancer progression. Studies have shown that the A2A2 genotype is associated with elevated IL-1β levels [30,33], which could contribute to chronic inflammation, a key factor in cancer development. Additionally, alcohol consumption in individuals with the A2A2 genotype has been shown to be associated with advanced CRC stages. Alcohol is a known risk factor for CRC, and, in this context, it may accelerate cancer progression by modulating the GSK3β/β-catenin pathway, further promoting tumorigenesis and advancing CRC development [34].

Additionally, the association between the A2A2 genotype and advanced CRC may be influenced by the interplay of genetic and environmental factors. The A2 allele may contribute to an inflammatory environment that accelerates tumor progression, particularly in individuals exposed to risk factors like alcohol consumption. Chronic inflammation driven by IL-1β and other cytokines can enhance tumorigenesis, suggesting that the A2A2 genotype could promote more aggressive CRC progression.

Finally, it was observed that the A1A2 genotype was associated with a decreased risk of CRC; a possible explanation for this is that the A2 allele, without the presence of the T allele, decreases the inflammatory process and thus the risk of CRC. Having only one A2 allele, however, reduces the possibility of the presence of the T allele; however, it is necessary to perform genotyping of the IL-1β variant to confirm this theory, since other publications have reported the association of the haplotypes of these two members of the IL-1 family with CRC, concluding that haplotypes formed with alleles associated with increased production of IL-1β, such as IL-1RN decrease alleles (≥4 repeats), are associated with neoplasms [30].

Regarding other alleles of the *IL-1RN* gene, a case–control study described that the A3 allele is associated with CRC. The study also reported a higher frequency of the A1 allele in patients with early-stage CRC compared to those with advanced stages [31]. The frequency of the A3 allele observed in the present study was low (0.7%) and was only present in one group of patients; therefore, a statistical analysis was not performed with this allele.

### 4.4. Variants in the IL-1β Gene

In the association study of the rs1143627 variant, we observed that the TC genotype was significantly associated with CRC, whereas the CC genotypes were associated with age at diagnosis of less than 50 years and overweight status. These results are consistent with previous research that has linked this variant with triple-negative breast cancer, as well as with clinical characteristics such as age younger than 40 years and hormone receptor (HR and ER) expression [12]. In addition, this variant has also been described to be associated with prostate cancer [35]. These findings suggest that the rs1143627 variant may play an important role in predisposition to various types of cancer, including CRC, and that its influence may manifest differently depending on the demographic and clinical characteristics of patients.

On the other hand, we observed that the rs16944 variant, specifically the T allele, was associated as a risk factor for CRC, while the C allele was also identified as a risk factor. However, in a previous study, it was reported that this variant was not associated with CRC in a population from Romania [36]. In addition, this variant has shown an association with cervical cancer in Asian women [37]. These findings suggest that the rs16944 variant may play a variable role in cancer susceptibility, depending on population-specific ethnic and genetic factors.

### 4.5. Haplotypes of Polymorphisms in IL-1β

In the present study, the *IL-1β* haplotype was shown to be in linkage disequilibrium. The above is in consistent with what was previously described [30], where it was observed that different polymorphisms in *IL-1β* are linked, among these the rs16944, and that the haplotype between allele 1 of VNTR IL-1RN and allele C of rs16944 is associated with stage II CRC. However, there is controversy in this regard; some authors emphasize that polymorphisms in *IL-1β* and *IL-1RN* are not in linkage disequilibrium. One study reported that the rs1143627 and rs16944 variants are in linkage in the Korean population [38], without being linked to the 89bp VNTR in *IL-1RN*. Another, similar study, in patients with keratoconus in the Korean population, observed that the rs1143627 and rs16944 variants are linked [35].

### 4.6. Plasma Levels of IL-1β Protein

IL-1β levels were not associated with CRC in the present study, which is consistent with a previous study [36] that found no association in serum IL-1β levels between 24 CRC patients and a control group. However, a recent study [37] reported significantly higher IL-1β protein levels in gastroesophageal cancer and squamous cell carcinoma.

### 4.7. In Silico Results

Our in silico results suggest that the rs1143627, rs16944, and rs2234663 variants are located at key sites for gene regulation, indicating their possible functional involvement in gene expression. These findings support the notion that these variants may play a crucial role in the regulation of gene expression, particularly in contexts involving transcription factor-mediated signaling pathways or chromatin accessibility. In a recent study [39], rs1143627 and rs16944 variants were reported to be associated with increased putamen volume in the left cerebral hemisphere, suggesting their possible involvement in brain structure development.

In mRNA expression analysis, we observed an overexpression of *IL-1β* and *IL-1RN* genes in CRC. These results are in agreement with a previous study [40], which also reported that *IL-1β* was overexpressed in CRC. The overexpression of *IL-1Β* suggests that this gene plays a proinflammatory role in the tumor microenvironment, thus contributing to cell proliferation and angiogenesis. In a recent study [41], it was described that overexpression of an *IL-1β/IL-1R* axis in CRC can influence epithelial–mesenchymal transition (EMT) and several carcinogenic processes, such as cell invasion and proliferation, among others.

On the other hand, *IL-1RN* showed considerable overexpression in CRC; these results are in agreement with a previous study [42], in which overexpression of this gene was also observed in thyroid cancer. On the other hand, a recent study [43] reported that *IL-1RN* is significantly underexpressed in oral squamous cell carcinomas (OSCCs) compared to normal tissues. However, despite its decreased expression in the early stages, it was observed that, in advanced cases of OSCC, *IL-1RN* expression is increased. The above findings suggest that, although *IL-1RN* acts as an *IL-1β* antagonist, its increase in tumor tissue could be an adaptive response to *IL-1β* -mediated inflammation.

Furthermore, the significant positive correlation in the co-expression of *IL-1β* and *IL-1RN* in READ suggests that both genes, although overexpressed in cancer, interact in an inflammatory cycle, as previously demonstrated [41], where a 1β/1RN axis regulates various aspects of CRC via autophagy. Thus, the above suggests that, while *IL-1β* potentiates tumor progression, *IL-1RN* overexpression may reflect an attempt by the organism to modulate that inflammation, although not enough to slow tumor progression. These dynamics highlight the complexity of inflammatory pathways in the tumor microenvironment.

When analyzing the clinicopathologic characteristics in relation to *IL-1β* expression, it was observed that *IL-1β* expression is associated with the pathologic T state in COAD, with higher expression in the T4 state and a lower expression in the T1 state. In READ, *IL-1β* expression was also associated with obesity, with higher levels observed in obese patients compared to normal-weight patients. These results are consistent with a previous study [44] in which this gene was found to be overexpressed in obese individuals. This suggests that *IL-1β*-mediated inflammation could be related to the patient’s metabolic context, where obesity could exacerbate inflammatory activity in the tumor microenvironment.

Likewise, *IL-1RN* expression also showed an association with BMI in READ, with higher levels in obese and lower levels in normal-weight individuals. This has also been observed in a previous study [45] where an increase in *IL-1RN* expression was reported in obese individuals. This relationship indicates that, although *IL-1RN* acts as an antagonist of *IL-1β*, its expression could be influenced by metabolic factors, suggesting a complex mechanism of regulation of inflammation in relation to the nutritional status of the patient.

Although no association between plasma IL-1β levels and CRC was found in this study, the discrepancy between mRNA overexpression and circulating IL-1β levels could be attributed to local regulation of IL-1β in tumor tissue. In the tumor microenvironment, IL-1β may be produced and regulated at the site of the tumor, leading to elevated mRNA expression without a corresponding increase in plasma levels. This suggests that, while plasma IL-1β levels might not reflect the local tumor activity, mRNA levels could serve as a more accurate marker of the biological activity of the tumor. On the other hand, the absence of a significant association between plasma IL-1β levels and CRC may indeed be attributed to the elevated mRNA levels, suggesting a potential discrepancy between systemic and local inflammatory responses.

The findings of this study highlight the potential relevance of *IL-1RN* and *IL-1β* gene variants and their expression patterns in the pathogenesis of CRC. The identification of regulatory variants associated with CRC could contribute to the development of genetic biomarkers for early detection or risk stratification in Mexican populations. Furthermore, the observed overexpression of *IL-1RN* and *IL-1β* in CRC tissues underscores their potential as therapeutic targets.

One limitation of this study is its population stratification. Although Mexican individuals of Mexican origin up to the third generation were included, genetic markers were not used to confirm this ancestry, which could introduce some bias. However, the association study identified novel findings on variants and their relationship with CRC.

Another important limitation is the lack of functional validation of the findings obtained in silico. It is considered relevant to perform experimental studies to evaluate our results on the expression patterns of *IL-1β* and *IL-1RN* genes specifically in the Mexican population. Likewise, it is crucial to experimentally confirm the regulatory function of the variants analyzed.

## 5. Conclusions

*IL-1RN* (rs2234663) and *IL-1β* (rs1143627, rs16944) variants were associated with clinicopathologic features of CRC and clinicopathologic features of the disease. However, haplotype analysis was not associated with CRC, and plasma IL-1β levels were not associated with CRC. In silico analysis suggests that these variants are located in regulatory regions of the genes, which may influence their expression. In addition, increased expression of *IL-1RN* and *IL-1β* genes was observed in CRC compared to normal samples, and both genes showed a positive correlation in this pathology.

## Figures and Tables

**Table 1:** Sociodemographic characteristics of the study groups.

	Patients (n = 230)		Reference (n = 256)				*p*-Value
Age Years ($\bar{x}$ ± SD)	59.4 ± 12.1		59.0 ± 11.9				0.379 *
	N	%	n	%	OR	IC (95%)	
<50 years	(45)	20	(60)	23			
≥50 years	(185)	80	(196)	77	1.25	(0.8–19.4)	0.354 **
Sex							
Female	(113)	49	(173)	68			
Male	(117)	51	(83)	32	1.2	(0.7–2.1)	0.44 **
Tobacco							
Yes	(113)	49	(36)	14	3.3	(1.6–6.5)	<0.001 **
No	(117)	51	(220)	86			
Alcohol							
Yes	(116)	50.4	(33)	13	4.4	(2.1–8.9)	<0.0001 **
No	(114)	49.6	(223)	87			

$\bar{x}$ (Sample Mean), SD (Standard Deviation), OR (Odds Ratio from regression analysis), * Student’s *t*-test; ** χ^2^ test.

**Table 2:** Clinicopathological characteristics of CRC patients.

CRC Patients (230)							
		(n)	(%)			(n)	(%)
Location	Colon	(80)	35	Lymph node metastasis	Positive	(96)	42
	Recto	(150)	65		Negative	(134)	58
Personal Pathological History	No	(135)	59	Metastasis	Yes	(125)	54
	DM-HAS	(66)	29		No	(105)	46
	Diverticula/Hemorrhoids	(29)	12	Chemotherapy Response	Yes	(82)	36
Stage	I–II	(36)	16		No	(148)	64
	III–IV	(192)	84	Carcinoembryonic Antigen (0–5 μg/L)	Normal	(76)	33
Adenocarcinoma Histology	Differentiated	(61)	27		Elevated	(154)	67
	Moderately differentiated	(142)	62				
	Not differentiated	(27)	11				

DM (Diabetes mellitus), HAS (systematic arterial hypertension).

**Table 3:** Allelic and Genotypic Distribution of the Studied Variants.

Variant	Genotypes	Patients (n = 230)		Reference (n = 256)		OR	(95% CI)	*p*-Value
rs2234663		(n)	%	(n)	%			
	*A1A1*	(104)	45.0	(85)	33.2	1.5	(1.50–1.04)	0.027
	*A2A2*	(50)	22.0	(55)	21.4	1.0	(0.65–1.5)	0.945
	*A1A2*	(73)	31.7	(111)	43.0	0.6	(0.41–0.88)	0.008
	*A1A3*	(2)	0.9	(0)	0			
	*A1A4*	(0)	0	(2)	1.0			
	*A2A3*	(1)	0.4	(0)	0			
	*A2A4*	(0)	0	(1)	0.4			
	*A2A5*	(0)	0	(2)	1.0			
	Alleles (2n = 460)			Alleles (2n = 512)				
	*A1*	(283)	61.5	(283)	55.3	1.2	(1.01–1.67)	0.048
	*A2*	(174)	37.8	(224)	43.7	0.78	(0.60–1.01)	0.060
	*A3*	(3)	0.7	(0)				
	*A4*	(0)		(3)	0.6			
	*A5*	(0)		(2)	0.4			
	Model							
Codominant	A1/A1	103	44.8%	84	32.8%	1.00		
	H *	75	32.6%	118	46.1%	1.95	(1.08–3.50)	0.046
	A2/A2	52	22.6%	54	21.1%	1.95	(0.99–3.83)	
Dominant	A1/A1	103	44.8%	84	32.8%	1.00		
	H-A2/A2	127	55.2%	172	67.2%	1.95	(1.14–3.32)	0.013
Recesive	A2A2	52	22.6%	54	21.1%	1.38	(0.76–2.52)	0.29
	A1/A1-H	178	77.4%	202	78.9%	1.00		
rs1143627								
	Genotypes	(n = 230)	%	(n = 220)	%			
	TT	(54)	23	(64)	29	1.0 *		
	TC	(143)	62	(112)	51	1.5	(1.1–2.2)	0.02
	CC	(33)	15	(44)	20			
	Alleles (2n = 460)			Alleles (2n = 440)				
	C	(251)	0.54	(240)	0.54			0.94
	T	(209)	0.46	(198)	0.46			0.94
	Model							
Dominant	TT	54	23	64	29	0.67	(0.37–1.22)	0.19
	TC + CC	176	77	156	71			
Recesive	CC	33	15	44	20	1.04	(0.522.06)	0.91
	TT + TC	197	85	179	80			
rs16944								
Model	Genotype	(n = 230)	%	(n = 247)	%			
	*CC*	(61)	27	(73)	30			
	*CT*	(132)	57	(129)	52	0.68	(0.38–1.23)	0.4
	*TT*	(37)	16	(45)	18	0.92	(0.422.03)	
	Alleles (2n = 460)			Alleles (2n = 494)				
	T	(254)	0.55	(215)	0.44	1.6	(1.2–2.0)	0.0003
	C	(206)	0.45	(279)	0.56	0.6	(0.5–0.8)	0.0003
	Model							
Dominant	CC	61	27	73	30	0.73	(0.42–1.28)	0.28
	CT + TT	169	73	174	70			
Recesive	TT	37	16	45	18	1.17	(0.49–2.36)	0.65
	CC + CT	98	42	118	47			

OR (Odds Ratio from regression analysis), CI (Confidence Interval). * includes A3, A4, and A5 alleles.

**Table 4:** Variants and their association with the clinical and pathological features in the CRC group.

Variant	Genotype	Variable	OR	(95%CI)	*p*-Value
rs2234663	A1A1	Stage I–II with diagnosis less than 5 years old	2.6	(1.1–5.8)	0.016
	A2A2	Stage III–IV and ethanol consumption	10	(1.3–38.1)	0.007
rs1143627	CC	Age (<50 years)	2.3	(1.1–5.6)	0.046
rs16944	CT	Hemorrhoids/Diverticula	2.7	(1.1–7.1)	0.041
	TT	Gastric toxicity	1.8	(1.1–3.3)	0.040
	TCCC	Stage III–IV with lymph nodes-metastasis	3.1	(1.3–7.9)	0.01

OR (Odds Ratio from regression analysis), CI (Confidence Interval).

**Table 5:** Haplotypic distribution among the study groups.

Haplotype		Frequency			OR	95%CI	*p*-Value
rs2234663 *	rs1143627	rs16944	CCR	Reference			
A1	T	C	0.2145	0.2198	0.9	0.479–1.850	1
A2	T	C	0.1291	0.2215	0.5	0.250–1.122	0.136
A1	C	T	0.1541	0.1946	0.7	0.358–1.580	0.572
A2	C	T	0.0918	0.145	0.6	0.250–1.476	0.375
A1	C	C	0.1241	0.0811	1.5	0.611–4.019	0.479
A1	T	T	0.1182	0.0631	2.1	0.768–5.937	0.216
A2	T	T	0.0838	0.0397	2	0.607–7.167	0.371

OR (Odds Ratio from regression analysis), CI (Confidence Intervals). * A3, A4, and A5 alleles were not included in the analysis because of their low frequency.

**Table 6:** Plasma levels of IL-1β in the study groups.

Group	(n)	Mean	SD	*p*-Value
CCR	48	103.81	19.26	0.47
Reference	34	101.39	2.81	
CCR				
I–II Stage	9	99.54	0.96	0.46
III–IV Stage	39	104.94	21.56	

SD (Standard Deviation).

**Table 7:** Expression of *IL-1β*, and *IL-1RN* in both COAD and READ.

	*IL-1β*		*IL-1RN*	
	COAD	READ	COAD	READ
Average	56.1	38.7	36.1	21.8
Median	20.5	17.2	17.6	12.1
	Normal	Normal	Normal	Normal
Average	15.6	34.4	7.4	10.8
Median	8.4	16	4.7	7.9
Lon2 FC	1.29	0.10	1.90	0.62
*p*-Value	6.8 × 10^−4^	8.3 × 10^−1^	1.7 × 10^−12^	1.5 × 10^−2^

**Table 8:** Clinical and Pathological Characteristics Related to *IL-1β*, and *IL-1RN* Expression.

			Average	Median	SD	*p*-Value *
*IL-1β*						
COAD	Stage	n				
	T1	6	12.5	11.5	3.4	1.70 × 10^−3^
	T2	44	38.2	30.6	32.9	
	T3	191	37.6	16.7	62.3	
	T4	37	119.7	20.5	456.9	
READ	BMI	n				
	Normal	14	22.4	10.4	22.4	2.00 × 10^−2^
	Under Weight	4	33.4	25.6	25.4	
	Obesity	14	69.5	19.9	138.5	
	Overweight	35	38.1	17.6	59.4	
*IL-1RN*						
READ						
	BMI	n				
	Normal	14	17.1	9.8	16.3	3.40 × 10^−2^
	Under Weight	4	19.7	21.9	8.8	
	Obesity	14	33.8	13.4	46.9	
	Overweight	35	21.8	11	22.4	

SD (Standard Deviation), * ANOVA test.

## Data Availability

Data and materials are available in the article.
